# Supplementary material for: The Sigma-1 Receptor Exacerbates Cardiac Dysfunction Induced by Obstructive Nephropathy: A Role for Sexual Dimorphism
Source: Biomedicines. 2024 Aug 20;12(8):1908. doi: 10.3390/biomedicines12081908 (PMC11351121; doi:10.3390/biomedicines12081908)
Supplement: Supplementary file 1 [file biomedicines-12-01908-s001.zip › Supplementary Table S1.pdf]

Supplementary Table S1. Primer pairs used in RT-qPCR

Symbol	Name	Forward primer (5´->3´)	Reverse primer (5´->3´)
Gapdh	Glyceraldehyde 3-phosphate dehydrogenase	GAAGGGCTCATGACCACAGT	TGCAGGGATGATGTTCTGGG
Ngal	Neutrophil gelatinase-associated lipocalin	GCCCTGAGTGTCATGTGTCT	GAACTGATCGCTCCGGAAGT
Anp	Atrial natriuretic peptide	AGCTTGGTCACATTGCCACT	AGAGGCAAGACCCCACTAGA
Bnp	Brain natriuretic peptide	ATCTCAAGCTGCTTTGGGCA	ACAACTTCAGTGCGTTACAGC
Acta2	Actin alpha 2, smooth muscle, aorta	AGCCATCTTTCATTGGGATGG	CCCCTGACAGGACGTTGTTA
Sigmar1	Sigma non-opioid intracellular receptor 1	GGTGAAGGGAAAGAGGAGCTG	TAGCAGGGGAGTGGAATGGA
Col1a1	Collagen, type I, alpha 1	GCTTCACCTACAGCACCCTT	GTCCGAATTCCTGGTCTGGG
